# Correction: Characterization of chitosan- and β-cyclodextrin-modified forms of magnesium-doped hydroxyapatites as enhanced carriers for levofloxacin: loading, release, and anti-inflammatory properties

**DOI:** 10.1039/d4ra90068e

**Published:** 2024-06-06

**Authors:** May N. Bin Jumah, Sarah I. Al Othman, Awatif Abdulaziz Alomari, Ahmed A. Allam, Mostafa R. Abukhadra

**Affiliations:** a Biology Department, College of Science, Princess Nourah bint Abdulrahman University Riyadh Saudi Arabia; b Zoology Department, Faculty of Science, Beni-Suef University Beni-Suef Egypt; c Department of Biology, College of Science, Imam Mohammad Ibn Saud Islamic University (IMSIU) Riyadh 11623 Kingdom of Saudi Arabia; d Geology Department, Faculty of Science, Beni-Suef University Beni-Suef 65211 Egypt Abukhadra89@Science.bsu.edu.eg +2001288447189; e Materials Technologies and Their Applications Lab, Geology Department, Faculty of Science, Beni-Suef University Beni-Suef City Egypt

## Abstract

Correction for ‘Characterization of chitosan- and β-cyclodextrin-modified forms of magnesium-doped hydroxyapatites as enhanced carriers for levofloxacin: loading, release, and anti-inflammatory properties' by May N. Bin Jumah *et al.*, *RSC Adv.*, 2024, **14**, 16991–17007, https://doi.org/10.1039/d4ra02144d.

The authors regret that the one of the affiliations (affiliation a) was incorrectly shown in the original manuscript. The corrected list of affiliations is as shown here.

The funding information was also shown incorrectly in the acknowledgements section of the original manuscript. The corrected funding acknowledgement is as shown below.

This work was funded by the Deanship of Scientific Research at Princess Nourah bint Abdulrahman University, through the Research Groups Program Grant no. (RGP-1444-0035)(2).

The Royal Society of Chemistry apologises for these errors and any consequent inconvenience to authors and readers.

## Supplementary Material

